# The Sterol Trafficking Pathway in *Arabidopsis thaliana*

**DOI:** 10.3389/fpls.2021.616631

**Published:** 2021-05-26

**Authors:** Krishna Kumar, Holly C. Gibbs, Alvin T. Yeh, Lawrence R. Griffing

**Affiliations:** ^1^Molecular and Environmental Plant Sciences Interdisciplinary Program, Texas A&M University, College Station, TX, United States; ^2^Department of Biomedical Engineering, Texas A&M University, College Station, TX, United States; ^3^Microscopy and Imaging Center, Texas A&M University, College Station, TX, United States; ^4^Department of Biology, Texas A&M University, College Station, TX, United States

**Keywords:** endocytosis, sterol uptake, sterol transport, ER transport, nuclear envelope, oil body

## Abstract

In plants, the trafficking mechanisms by which sterols move through the plant and into target cells are unknown. Earlier studies identified endosomes as primary candidates for internalization of sterols in plants, but these results have come into question. Here, we show that in elongating root cells, the internalization of sterol occurs primarily by a non-endocytic mechanism. Added fluorescent sterols [dehydroergosterol (DHE) and BODIPY-cholesterol (BCh)] do not initially label endosomes identified by fluorescent protein markers or by internalized FM4-64. Instead, the nuclear envelope, an organelle not associated with the endocytic pathway but part of the endoplasmic reticulum (ER), becomes labeled. This result is supported by experiments with the inducible overexpression of auxilin-2-like protein (AUX2 line), which blocks most endocytosis upon induction. Internalization and nuclear envelope labeling still occur in induced AUX2 cells. Longer-term incubation labels the oil body, a site involved in sterol storage. Although the first site of localization, the nuclear envelope, is part of the ER, other domains of the ER do not accumulate the label. The trafficking pathway differs from vesicular endocytosis and points toward a different pathway of sterol transport possibly involving other mechanisms, such as ER–plasma membrane contact sites and cytoplasmic transport.

## Introduction

The movement of sterols, in particular cholesterol, in the plant is of interest for a variety of reasons. First, although the claim of being “cholesterol-free” is made in the United States for many plant-based food products (based on Food and Drug Administration guidelines), plants do indeed have cholesterol (Behrman and Gopalan, 2005), and the genetics of the pathway for the synthesis of cholesterol have recently been outlined (Sonawane et al., 2016). Although cholesterol is about 6% of the sterol content in *Arabidopsis*, it can be higher in other plants, such as in *Solanaceous* species, where cholesterol is commonly esterified and forms toxic steroidal alkaloids through the GLYCOALKALOID METABOLISM (GAME9) pathway (Cardenas et al., 2016). Second, some of the cholesterol that is made in the shoot is exported to the phloem, where it is translocated to the root (Devarenne et al., 2002; Behmer et al., 2013). Why plants have this kind of selective transport of cholesterol is unknown, but phloem-feeding insects rely on this pathway for the production of the molting hormone, ecdysone, not being able to make cholesterol themselves (Behmer et al., 2013). Altering the movement of cholesterol in plants could have a profound impact on the life cycle of phloem-feeding insects and is therefore of great agronomic interest. Third, many of the embryo-lethal mutants in *Arabidopsis*, such as Fackel and Hydra2, are mutations in the Δ^8,14^ sterol C-14 reductase, which is in the precursor pathway leading to the production of cholesterol, stigmasterol, and brassicasterol and cannot be chemically compensated by brassinolides alone (Clouse, 2002). Fourth, mutations in later stages of the sterol biosynthetic pathway show mis-localization of PIN2, the auxin transporter, and have defects in cell plate formation (Men et al., 2008).

The change in the localization of PIN2 and the change in endocytosis of other proteins and endocytic labels that accompany these mutations in sterol biosynthesis have been interpreted as evidence for the vesicular movement of sterols from the plasma membrane (PM) to the endosomal compartment via endocytosis (Boutte and Grebe, 2009; Stanislas et al., 2014). Early work (Grebe et al., 2003) supporting this interpretation, using *in vivo* labeling with the fluorescent polyene antibiotic, filipin, which binds sterols in the PM and also labels endocytic structures, has been reevaluated. Filipin apparently inhibits endocytosis (Boutté et al., 2011) and, when added to root hairs, causes aberrant endocytic structures to form (Oveĉka et al., 2010). This is probably a consequence of the formation of sterol–filipin aggregates in the PM that perturb membrane function (Oveĉka et al., 2010).

Hence, the importance of sterols for the process of endocytosis does not necessarily mean that vesicular endocytosis is the main mechanism for internalization of PM sterols. There are other potential mechanisms for the delivery of sterols to endosomes including transport by cytoplasmic sterol-binding proteins (Iaea et al., 2017) and transport to recycling endosomes via the *trans-*Golgi network and transport to endosomes via endoplasmic reticulum (ER)-to-endosome membrane contact sites (MCS) (Friedman et al., 2013). If vesicular endocytosis is not the mechanism for internalization of sterol, the other main candidate for sterol (and other lipid) transport from the membrane into the cell in plants is putative ER–PM MCS (Li-Beisson et al., 2013). Although ER–PM MCS or anchor sites have been visualized using persistency mapping (Sparkes et al., 2009) and molecular components of the MCS have been identified (Wang et al., 2014; Levy et al., 2015; Perez-Sancho et al., 2015), the function of the ER–PM MCS in plants has not been elucidated. Tantalizing evidence that the specialized ER–PM MCS occurring in the plasmodesmata might be involved in sterol biosynthesis or transport comes from the observations that plasmodesmata are enriched in sterols (Grison et al., 2015) and that plasmodesmatal reticulons, ER proteins involved in the tubulation of the ER (Sparkes et al., 2011), can bind to sterol methyl transferase 1 (SMT1) as a partner (Kriechbaumer et al., 2015). The strongest evidence that ER–PM MCS are engaged in sterol transport, however, comes from other systems, such as yeast, where proteins involved in sterol transport, other than oxysterol-binding proteins, have been identified (Gatta et al., 2015).

Therefore, we undertook an investigation into the mechanism of sterol entry into the plant cell to determine whether vesicular or non-vesicular uptake is the primary route of entry of sterols. For this, we use the fluorescent sterols BODIPY-cholesterol (BCh) and dehydroergosterol (DHE). Our data are more consistent with a non-vesicular entry of sterols than with an endocytic vesicular uptake of sterols. The immediate target of internalization is a subdomain of the ER, the nuclear envelope (NE), which labels within 5 min of exposure to fluorescent sterols. Interestingly, there is no accumulation of sterols in other subdomains of the ER, a result which may be expected because even though it is the primary site of sterol biosynthesis within the cell (Hartmann, 2004), there is proportionally less sterol in the ER than in other endomembrane compartments (Moreau et al., 1998b).

## Materials and Methods

### Plant Materials and Growth Conditions

The wild-type and transgenic seeds of *Arabidopsis thaliana* were surface-sterilized with 70% ethanol and planted on 1/2 strength Murashige and Skoog (Caisson Labs, United States) 1% agar (Sigma-Aldrich, United States) medium containing vitamins and phosphates, and the pH was adjusted to between 5.6 and 5.8. Following cold, dark treatment for 48 h in Petri dishes, the seedlings were grown for 4–5 days at 22° under continuous white light in a vertical position and treated for analysis. The following homozygous transgenic *Arabidopsis* lines tagged with fluorescent fusions were used in this study: RFP and GFP targeted to NE (SUN-RFP and SINE2-GFP) (Zhou et al., 2014, 2015), an mCherry-tagged PM marker (NPSN12) (Geldner et al., 2009), an mCherry-tagged late endosomal marker (RabF2a-mCherry) (Geldner et al., 2009), an mCherry-tagged *trans-*Golgi network/early endosomal marker (VTI12-mCherry) (Geldner et al., 2009), and mCherry-HDEL (Cheng et al., 2017). The β-estradiol-inducible auxilin-2-like (AUX2) *Arabidopsis* line was from the Friml lab (Adamowski et al., 2018).

### BCh, DHE, FM4-64, Filipin, and Nile Red Labeling

BODIPY-cholesterol (TopFluor^®^, Avanti Polar Lipids, United States) and DHE (Sigma-Aldrich, United States) were prepared as a stock solution with a slight modification from that previously described (Holtta-Vuori et al., 2008). BCh was dissolved in 100% ethanol to 1.7 mM in the stock solution and DHE at 10 mM in the stock solution and combined with methyl-β-cyclodextrin (MβCD) (Sigma-Aldrich, United States) at a molar ratio of 1:3 to a final concentration of 10 μM BCh or DHE and 30 μM MβCD. The probe was either sonicated for 30 min and vortexed for 15 min before use or just vortexed for 30 min before use. The styryl dye FM4-64 (Invitrogen, United States) at a final concentration of 4 μM was used for dual labeling with BCh. Filipin (Sigma-Aldrich, United States) was made up of a DMSO stock solution (15 mM) and diluted 1:500 in water for treatment. For oil body staining, seedlings were incubated in 2 μg/ml Nile Red (Sigma-Aldrich, United States) solution (made from 2 mg/ml stock in acetone).

### Confocal Microscopy and Image Analysis

Confocal laser scanning microscopy imaging was performed on intact *Arabidopsis* wild-type (Col-0) and transgenic seedlings grown on agar plates. Whole seedlings were stained for the time indicated. Imaging was done on an Olympus FluoView 1000 confocal imaging system equipped with 60X, 1.2 NA water immersion objective. Images and time-lapse videos for analysis were taken using a 20 μs/pixel dwell time. BCh (λex = 497 nm, λem = 507 nm, Holtta-Vuori et al., 2008) was examined with the 488 nm line of an argon ion laser, and fluorescence was recorded between 500 and 530 nm. mCherry and RFP fusion proteins were examined with a 543 nm He–Ne laser and fluorescence recorded between 585 and 685 nm. FM4-64 labeling also used the 543 nm He–Ne laser and fluorescence recorded at 640–700 nm (Bolte et al., 2004). Analysis and post-processing of images were performed with ImageJ (Schneider et al., 2012), FIJI (Schindelin et al., 2012), and Adobe Photoshop^®^ as well as Adobe Illustrator^®^ (CS 6, Adobe Systems, San Jose, CA, United States). The relative intensity of NE was calculated by subtracting the cytoplasmic integrated density per unit area from the NE integrated density per unit area.

### Multiphoton Laser Microscopy

Fluorescent observation of DHE was carried out with multiphoton microscopy. For multiphoton imaging, 10 fs pulses centered at 800 nm with a bandwidth of 133 nm were pre-compensated and coupled to a 20X, 1.0 NA objective with x–y scanning mirrors. An average power of 25 mW was used on live samples. Fluorescence signals were separated by a dichroic long-pass mirror at 430 nm. Wavelengths below 430 nm passed through a BG-39 filter, and wavelengths above 430 nm passed through a bandpass filter centered at 450 nm with a bandwidth of 60 nm to separate the DHE fluorescence and cellular autofluorescence that were subsequently detected with photomultiplier tubes.

## Results

### BCh and DHE Track Endogenous Sterols

**FIGURE 1:**
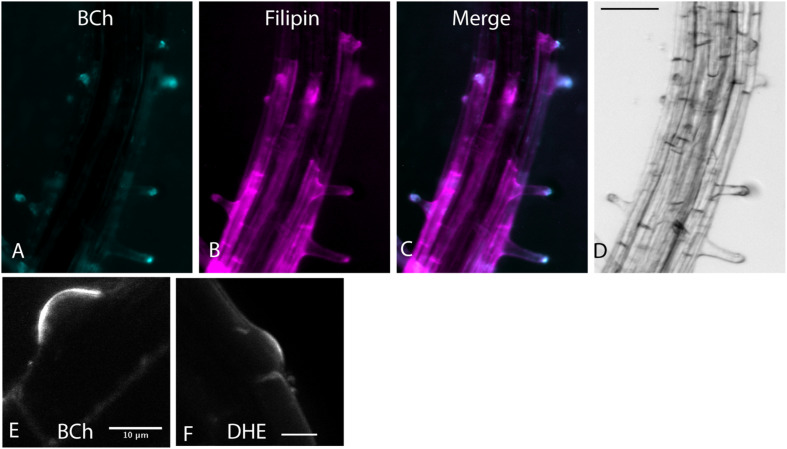
Root hair tip localization of BCh, filipin, and DHE in 5-day-old *Arabidopsis* seedlings labeled for 60 min with both dyes and 30 μM MβCD. **(A)** 10 μM BCh localization primarily in the tips of the root hairs. **(B)** 15 μM filipin labeling of root hair tips and along the PM of epidermal cells in the mature regions of the root. **(C)** Merged image of **(A** and **B)**. **(D)** Bright-field image of the root. Scale bar = 100 μm. **(E)** Emergent root hair labeled with BCh for 20 min, similar to that shown in the upper part of **A**. Scale bar = 10 μm. **(F)** Emergent root hair at the base of an epidermal cell labeled for 20 min with DHE. Scale bar = 10 μm.

BODIPY-cholesterol and DHE (10 μM) label a region of native sterol accumulation, the tip of the root hair. Sterols accumulate there (Oveĉka et al., 2010), as indicated by their staining with filipin. As shown in Figure 1, filipin, BCh, and DHE label the tip of emerging root hairs, supporting the hypothesis that they are effective tracers of endogenous sterol distribution.

### BCh and DHE Label the NE and PM of Elongating Cells of the Root

**FIGURE 2:**
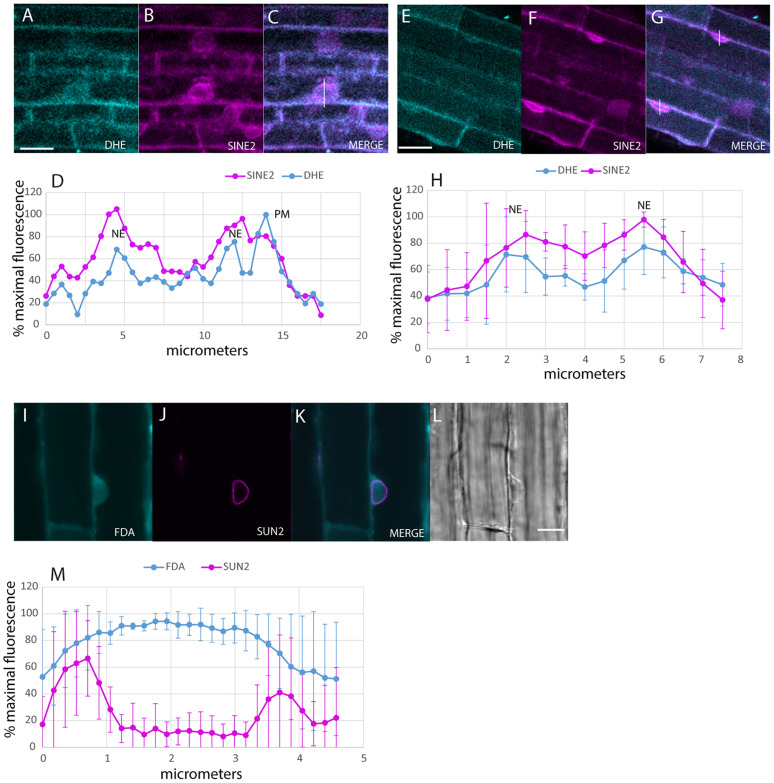
Labeling of the NE with DHE in elongating root cells. **(A–C)** Seedlings expressing SINE2-GFP were incubated for 30 min in DHE and fluorescence after multiphoton excitation was measured. **(A)** Emission at 405/20 nm for DHE. **(B)** Emission at 525/50 nm for SINE2-GFP. **(C)** Merged image of the DHE signal (cyan) and the SINE2-GFP signal (magenta). Scale bar = 20 μm. **(D)** Profile plot of line shown in **(C)**. PM indicates peak from PM. NE indicates peaks from the membranes of the NE. **(E–G)** Seedlings expressing SINE2-GFP were incubated for 30 min in DHE as in **(A)**, but the nuclei of these cells lie against the PM. **(E)** DHE signal. **(F)** SINE2-GFP signal. **(G)** Merged image. **(H)** Averages of profile plots of five similarly sized (smaller than the nucleus in **A–D**) nuclei show a bimodal distribution of intensity peaking at the NE. The error bars indicate standard deviation. **(I–L)** Seedlings expressing SUN-RFP were incubated for 30 min in the vital dye fluorescein diacetate to label the cytoplasm. **(I)** Fluorescein signal. **(J)** SUN-RFP signal. **(K)** Merged image. Scale bar = 10 μm. **(L)** Bright-field image. **(M)** Average of five profile plots of nuclei that lie against the PM, including the one shown in **(I–L)**. The error bars indicate standard deviation.

Uptake of the DHE into the NE after 30 min was detected with multiphoton microscopy (Figure 2). Colocalization was done in plants expressing SINE2-GFP, an NE protein (Zhou et al., 2014; Figures 2A–H). Line plots of the label across the nucleus produce a double-peaked profile for both DHE and SINE2-GFP, in larger, rounder nuclei (Figures 2A–D), as well as for more typical nuclear profiles, flattened against the PM (Figures 2E–H). Nuclei of plants expressing SUN-RFP also show a two-peaked profile, whereas the cytosolic dye, fluorescein diacetate, shows a broad single peak (Figures 2I–M) because it diffuses from the cytoplasm into the nucleoplasm.

**FIGURE 3:**
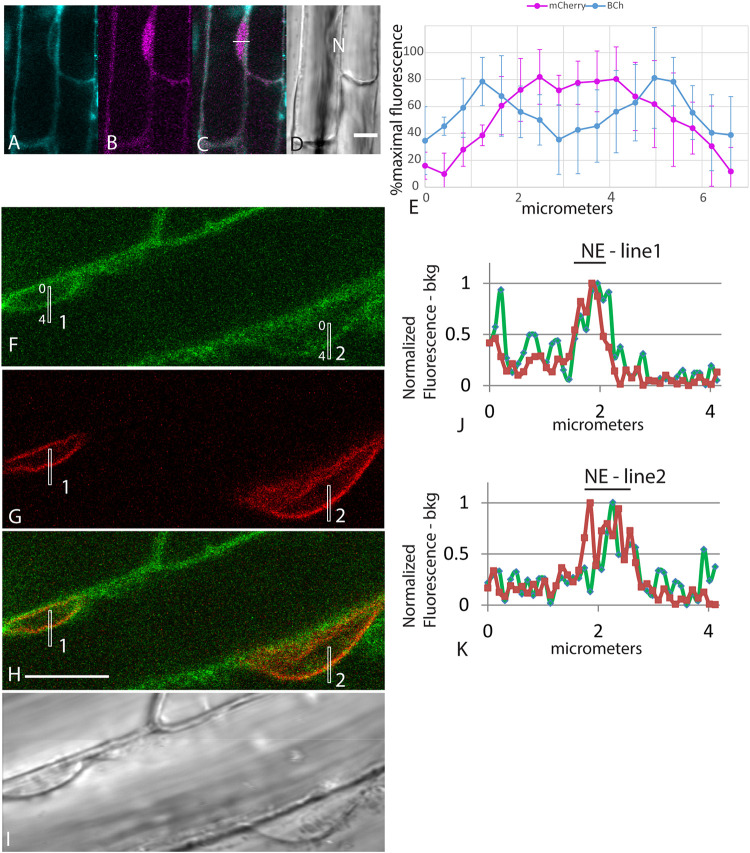
NE label with BCh. **(A–E)** Comparison of BCh staining after 25 min with expression of cytosolic mCherry. **(A)** BCh signal. **(B)** cytoplasmic mCherry signal. **(C)** Merged image of **(A** and **B)**, showing the line across which the profile was analyzed. **(D)** Bright-field image. N, nucleus. Scale bar = 10 μm. **(E)** Average line plot values (plus/minus standard deviation) of four nuclei lying flat against the plasma membrane, as in **(A–D)**. Note that mCherry has a broad peak and that BCh has two peaks at the edges of the nuclei. **(F–K)** BCh label after 30 min and colocalization of the label with the NE marker SUN-RFP. **(F)** BCh labels the PM and the NE of cells in the elongation zone of roots. **(G)** SUN-RFP expression in the NE of the cells shown in **(F)**. **(H)** Merged fluorescence micrographs **(F,H)**. Scale bar = 10 μm. **(I)** DIC image of the fluorescent region in **(F–H)**. **(J)** Fluorescence in the 4 μm line outlined in region 1 of **(F–H)** that crosses the NE. **(K)** Fluorescence in the 4 μm line in region 2 of **(F–H)**. In **(F** and **G)**, the fluorescence intensity in the BCh channel is green, while that in the SUN-RFP channel is red.

The accumulation of fluorescent sterol in the NE is also seen with BCh. Elongating root cells of plants expressing either SUN-RFP (Zhou et al., 2015) or cytoplasmic mCherry were treated for 30 min with BCh, and colocalization with the NE was seen (Figures 3A–K). In cytosolic mCherry-expressing plants, line profiles of the mCherry signal show a single broad peak, indicating that it labels the nucleoplasm as well as the cytoplasm, while line profiles of the BCh label show a two-peaked profile (Figures 3A–D). Line scans across two separate nuclei (Figures 3J,K) reveal that the peaks of SUN-RFP and BCh closely correspond at the NE.

**FIGURE 4:**
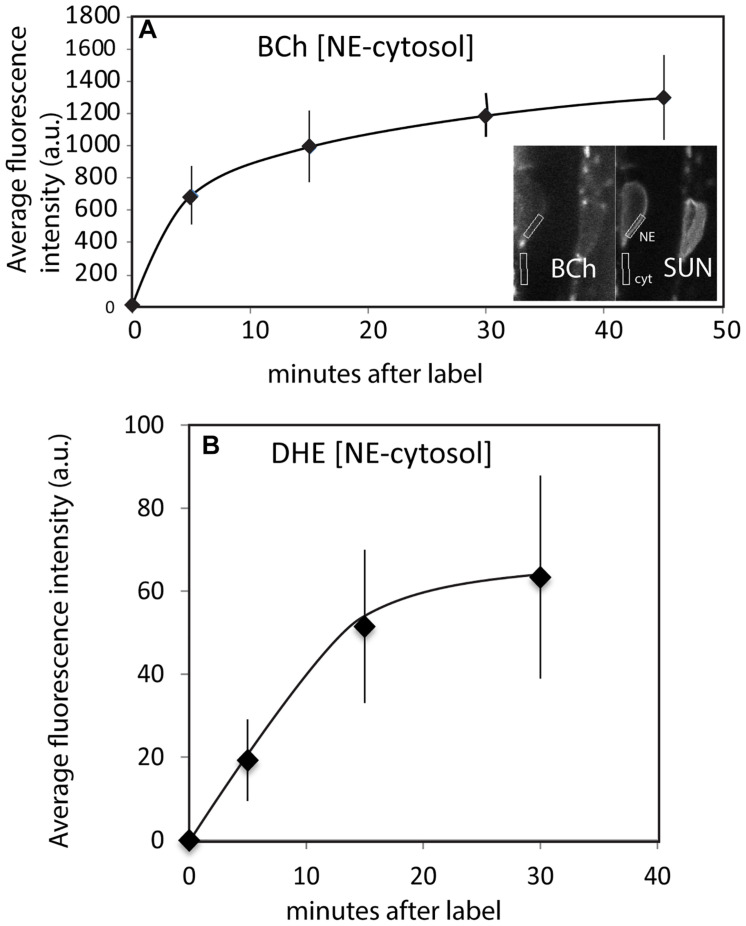
Uptake of BCh and DHE into the NE over time. **(A)** Average fluorescence intensity of BCh taken over a region containing the NE with an equivalent area of the adjacent cytoplasmic fluorescence subtracted in plants expressing SUN-RFP. Error bars are the standard deviation of five separate samples. Inset shows the identification of the NE and adjacent cytoplasm in the SUN-RFP channel. **(B)** Average fluorescence intensity of DHE uptake into NE in plants expressing SINE2-GFP. The fluorescence background in the cytoplasmic region has been subtracted. Error bars are the standard deviation of five separate cells.

The time course of internalization of the fluorescent sterols and their appearance in the NE is shown in Figures 4A,B. Labeling of the NE with DHE and BCh can be seen within 5 min. Labeling with both DHE and BCh reaches saturation fairly quickly, within 30 min. Some of the label may be in the thin cytoplasmic layer between the vacuole membrane and the NE. To correct for this, the uptake into the NE shown in Figure 4 was determined for BCh in cells expressing SUN-RFP and for DHE in cells expressing SINE2-GFP by outlining a region of the envelope marker expression for quantitation and subtracting the intensity of the sub-PM cytoplasmic label found in nearby regions (Figure 4B).

### BCh Label at the PM and Near the Cortical ER

**FIGURE 5:**
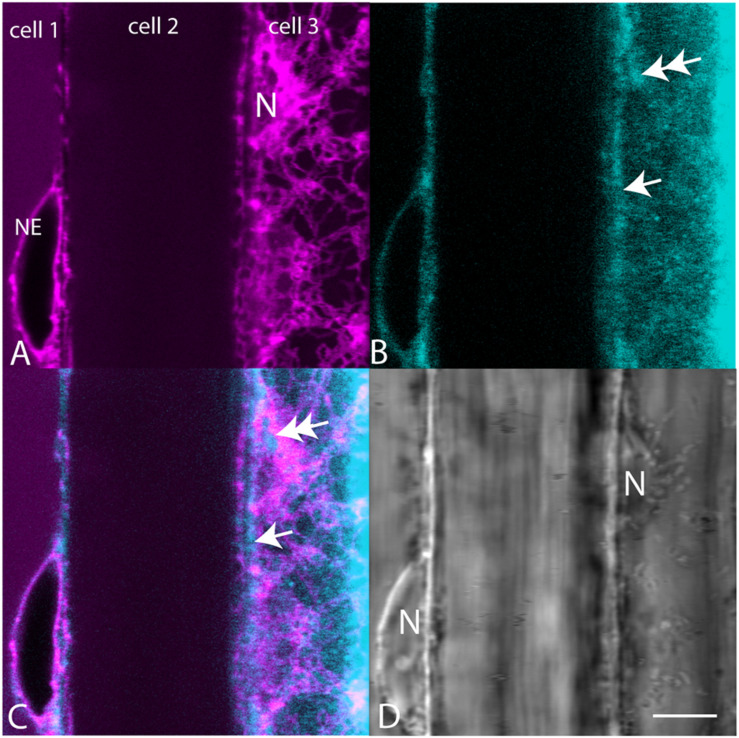
Comparison of mCherry-HDEL label (magenta) of the NE and cortical ER with BCh label (cyan, 16 h). **(A)** mCherry-HDEL label of the NE in cell 1 and the cortical ER and glancing optical section of the nucleus (N) in cell 3. **(B)** BCh label of the NE in cell 1 and diffuse label of the cortical region of the cytoplasm in cell 3. Double arrow points to the region on the glancing section of nucleus. Single arrow points to PM. **(C)** Merged image of **(A** and **B)**. **(D)** Bright-field image N, nuclei. The nucleus in cell 3 is in the glancing optical section. Scale bar = 10 μm.

**FIGURE 6:**
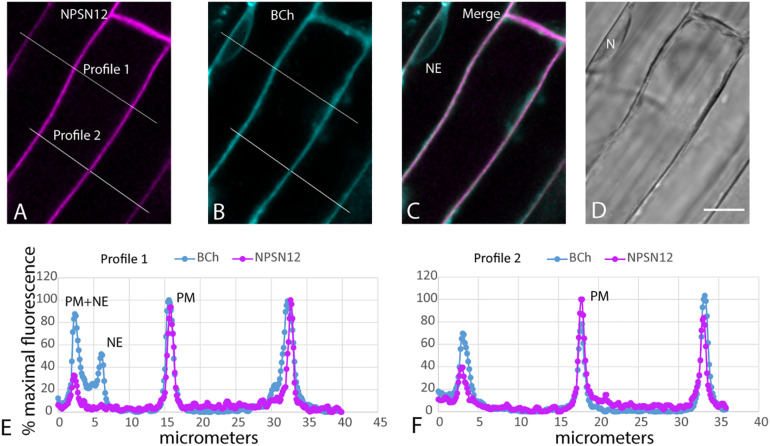
Label with BCh colocalizes with the PM and NE in elongating root cells. Five-day-old seedlings expressing NPSN12-mCherry were treated with BCh for 15 min. **(A)** PM marker NPSN12-mCherry fluorescence (magenta). **(B)** BCh signal (cyan). Line profiles in **(A** and **B)** are the same, and intensities are plotted in **(E** and **F)**. **(C)** Merged image, NE, nuclear envelope. **(D)** Bright-field image, N, nucleus. Scale bar = 10 μm. **(E)** Percentage of maximal fluorescence along line profile 1 in **(A)** NPSN12-mCherry (magenta) and **(B)** BCh (cyan). **(F)** Percentage of maximal fluorescence along line profile 2 in **(A)** NPSN12-mCherry (magenta) and **(B)** BCh (cyan). Colocalization at the PM is shown for two different cells at the center peak. NE peaks from **(B)** are shown as NE in **(E)**.

When plants expressing mCherry-HDEL in the ER were treated with BCh for short periods of time, no colocalization in the cortical ER was seen, and in an attempt to reveal any accumulation in the network, the plants were treated with BCh for 16 h (Figure 5). Even at these extended times, although there is a strong label of the NE in cell 1, there is little colocalization between the ER and the BCh in the cortical cytoplasm outside the NE in cell 3, where the cortical ER network is very clear. The label is at or near the plane of the PM, which is emphasized along the boundary between cell 2 and cell 3 (single arrow). A region containing a glancing optical section of the nuclear membrane (double arrows) also shows some BCh label in cell 3. Line profiles of cells from plants expressing NPSN12-mCherry, a PM marker, show colocalization of the BCh (30 min label) with the PM (Figure 6), as well as a label in the NE. The profiles also suggest that there is a cytoplasmic label in the third peak of profile 1 and the first peak of profile 2.

### BCh Does Not Label Early or Late Endosomes

**FIGURE 7:**
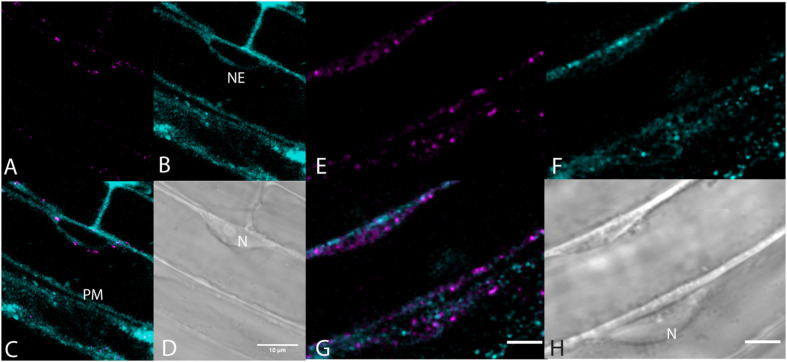
Lack of endosome labels, VTI12-mCherry, and RabF2a-mCherry, with BCh of the elongating root cells. **(A–D)** Structures labeled after 30 min of incubation in BCh (cyan) in plants expressing the early endosome/*trans-*Golgi marker, VTI12-mCherry (magenta). **(A)** Fluorescence of VTI12-mCherry organelles. **(B)** Fluorescence of BCh labels the NE and the PM. **(C)** Merged image of **(A** and **B)**, where magenta endosomes are outside the NE and in the cytoplasm. **(D)** DIC micrograph showing the nucleus (N). Scale bar = 10 μm. **(E–H)** Structures labeled with BCh (cyan) after 30 min in plants expressing RabF2a-mCherry (magenta). **(E)** Late endosomes labeled with RabF2a-mCherry. **(F)** BCh (cyan) labels the PM and non-endosomal punctae in the cytosol. **(G)** Merged image of **(E** and **F)** showing magenta endosomes that do not colocalize with the non-endosomal cyan punctae. **(H)** DIC image showing a glancing optical section of the nucleus (N). Scale bars in **(G)** and **(H)** = 5 μm.

Very little labeling of endosomes by BCh was seen when endosomes were visualized with fluorescent fusion protein markers, Rha1 (RabF2a)-mCherry (Geldner et al., 2009) for the late endosomes and VTI12-mCherry for the early endosomes/*trans-*Golgi (Geldner et al., 2009; Figure 7). The elongating roots of intact seedlings expressing these fusion protein markers were labeled with BCh for 5–30 min. In all cases, the BCh does not appreciably label endosomes. However, the BCh does label the NE and PM (Figures 7B,C).

**FIGURE 8:**
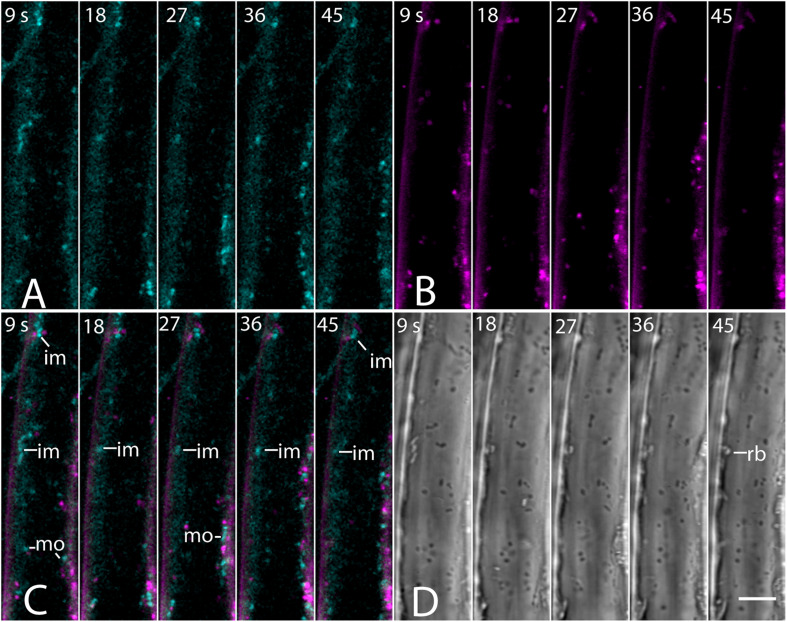
Labeling of elongating root cells with BCh (cyan, **A**) and FM4-64 (magenta, **B**) after 30 min of co-incubation. **(A)** Fluorescent structures associated with the PM and in the cytoplasm. **(B)** Red fluorescent, mostly motile, endosomes labeled with FM 4-64 after 30 min. **(C)** Merged image of **(A** and **B)** – punctate structures do not colocalize. BCh labels immobile (im) and motile (mo) structures. **(D)** Bright-field image. rb, refractile body. Images taken at 9 s intervals. Scale bar = 5 μm.

The absence of a BCh label in endosomes was confirmed with co-incubation of 5- to 7-day-old seedlings with 4 μM FM4-64 and 10 μM BCh for 30 min. Epidermal cells of the elongating region of the root were examined in near-tangential optical sections of the outer periclinal cytoplasm (Figure 8). An internal label with BCh showed little colocalization with FM4-64. Several of the regions labeled with BCh were relatively immobile (im, Figure 8C), although there were some moving punctae (mo, Figure 8C). Some of the immobile punctae are refractile in the bright-field image (Figure 8D, rb).

### Internalization in Induced AUX2 Overexpression

**FIGURE 9:**
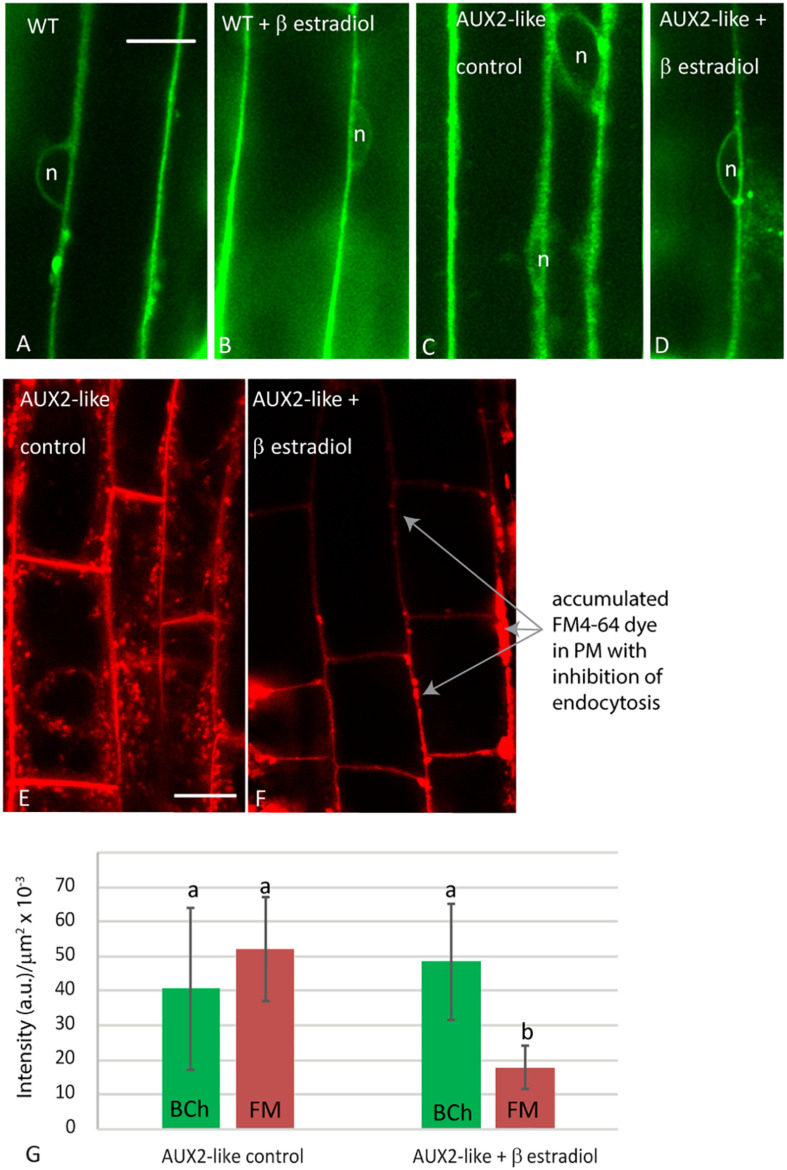
Analysis of uptake in AUX2 expression lines. Confocal micrographs of elongating root cells following **(A–D)** 30 min labeling with BCh in **(A)** the wild-type (WT) *Arabidopsis* line, **(B)** WT line pretreated with 9 mM β-estradiol, **(C)** the AUX2 line, and **(D)** the AUX2 line pretreated with 9 mM β-estradiol; **(E–F)** 30 min labeling with FM4-64 in **(E)** the AUX2 line and **(F)** the AUX2 line pretreated with 9 mM β-estradiol. **(G)** Quantitation of label intensity per square micrometer of cytoplasm in four to five different cells in three different plants. (b) column values <0.05p compared to (a) column values, n, nucleus. Scale bars = 10 μm.

To address the possibility that the absence of BCh in endosomes may be a consequence of a low but rapidly transported level of BCh in endosomes that is not detected with confocal microscopy, we examined the uptake of BCh after inhibiting endocytosis with the β-estradiol-inducible expression of AUX2 (Adamowski et al., 2018; Figure 9). After 30 min of incubation in BCh, the level and pattern of cytoplasmic label in elongating root cells do not change from those seen in wild-type plants (Figure 9A), in wild-type plants preincubated in β-estradiol (Figure 9B), in AUX2-like plants (Figure 9C), or in AUX2-like plants pretreated with β-estradiol (Figure 9D). In contrast, uninduced AUX2-like plants show normal levels of internalization of FM4-64 (Figure 9E) but show greatly reduced uptake in β-estradiol-induced AUX2-like plants (Figure 9F). As shown by Adamowski et al. (2018), when the internalization of the FM4-64 is inhibited, it accumulates in pockets and along stretches in the PM (Figure 9F). The quantification of internalization in β-estradiol-induced and uninduced AUX2-like plants shows significant reduction of internalization of FM4-64 in the induced AUX2 line, but no significant reduction of internalization of BCh in the induced AUX2 line (Figure 9G).

### Nature of the Punctate Label With BCh in Root Cells

The label with BCh near the PM and NE is somewhat discontinuous (Figure 7) and, when interior, punctate (Figure 8). BCh can be found in the highly refractile bodies (Figure 8D). Colocalization with Nile Red reveals that these refractile bodies are predominantly oil bodies. Following uptake, the BCh is probably esterified and BCh esters would accumulate in the lipid body. Long-term incubation in BCh has no effect on growth (Supplementary Figure 1).

## Discussion

Although filipin is not an accurate tracer of the physiological internalization of sterols (Oveĉka et al., 2010), it does accurately label sterol-enriched membranes in fixed tissue and surface sterols in living plants (Boutté et al., 2011). The root hair tip localization of filipin (Figure 1B) is also seen with BCh (Figure 1A). Likewise, when examined with multiphoton microscopy, DHE labels the tip of emerging root hairs, as does BCh (Figures 1A,E,F). This indicates that a modified sterol (BCh) and a foreign sterol (DHE) can be used to accurately trace local sterol distribution. DHE is an analog of ergosterol, a fungal sterol that acts as a signal for the plant cell to initiate a defense response (Tugizimana et al., 2014), so long-term (>1 h) treatments are not shown. In the presence of 10 μM BCh, on the other hand, plant growth is normal (Supplementary Figure 1).

As shown in Figures 2–4, BCh and DHE both quickly label the NE. The absence of both sterol probes in the nucleoplasm (Figures 2, 3) indicates that they do not simply diffuse through the cytoplasm and into the nucleus as occurs with fluorescein generated from fluorescein diacetate (Figures 2I–M) or cytoplasmic mCherry (Figures 3A–E). An initial (5–10 min) label does not occur in motile, streaming punctae but is found at the PM and the NE. Uptake into the NE is fast. The data do not exclude diffuse uptake into the cytoplasm, but when the level of cytoplasmic fluorescence is subtracted from NE fluorescence (Figure 4), the NE label appears higher than the cytoplasmic label and saturates over time. Furthermore, when a line plot of labeling intensity by BCh is compared with a marker for the NE, there is little label in the cytoplasmic region outside the region marked by the SUN-RFP, NE signal (Figures 3F–K).

In order to label cells with sterols, they had to be in the presence of the delivery agent, MβCD. Although MβCD has not been used previously in sterol uptake experiments in plants to our knowledge, it has been used to deplete sterols from plant membranes (Roche et al., 2008; Li et al., 2011). MβCD apparently dimerizes when it binds sterols (Lopez et al., 2013) and can either extract or supply sterols to a membrane.

The appearance of sterols in the NE is not so surprising when one considers that the concentration of cholesterol (Kemp and Mercer, 1968) and other sterols (Philipp et al., 1976) in the NE is higher than that in the ER in general. There are hypotheses for the function of the accumulation of sterols and other lipids in the NE in, for instance, yeast whereby their presence is postulated to be a storage mechanism for lipid during the elaboration and growth of the membrane that occurs during cytokinesis (Byrne, 2012; Witkin et al., 2012).

The rest of the ER does not accumulate sterols during their synthesis (Moreau et al., 1998a). The absence of sterols in the ER during their biosynthesis indicates that there is rapid transport out of the ER. This is consistent with the observations in Figure 5, showing that the ER does not accumulate BCh. The label that is in focus in the en face view of the cell cortex, which reveals the ER network, does not form a pattern (Figure 5B) but does label the NE in the cross section, cell 1 (Figure 5B), that is also labeled with the ER marker (Figure 5A) and in the glancing optical section (Figure 5B, double arrow). This non-patterned, but sometimes punctate, BCh label is mostly in the PM (Figure 6), although there is some label as well in regions of the cytoplasm (Figure 6E, third peak, and Figure 6F, first peak).

The label seen after 30 min of incubation in BCh does not colocalize with early or late endosomes, as assessed by markers for these organelles (Figures 7, 8). Although BCh does label some motile and immotile punctae at this time (Figures 7F, 8A), this label does not colocalize with an early endosome marker (Figures 7A–D) or a late endosome marker (Figures 7E–G). The continuous incubation of the cells with BCh over this time frame should provide a constant flow of membrane into both early and late endosomes were the BCh taken up endocytotically. Absence of BCh in the endocytic pathway was confirmed by the absence of colocalization with FM4-64 (Figure 8), a marker of membrane-bound endocytosis (Griffing, 2008). The presence of BCh would be expected were there endocytosis of sterols through a common, clathrin-mediated endocytic pathway.

However, if the endocytic delivery pathway were extremely transient, as is postulated for the delivery of sterols out of the ER upon their synthesis in the ER, then inhibiting endocytosis should rule it out. This was addressed by following the initial uptake of BCh in plants where endocytosis was inhibited with the induced overexpression of AUX2 protein (Adamowski et al., 2018). With the induction of overexpression of AUX2 protein with β-estradiol, endocytosis of FM4-64 is inhibited (Figures 9E–G). The aberrant accumulation of FM4-64 in regions of the PM under these conditions (Figure 9F) repeats the earlier work of Adamowski et al. (2018). The remaining level of the FM4-64 label quantified in Figure 8G may be the consequence of the accumulation of FM4-64 in PM domains that appear with inhibition of endocytosis. However, the uptake of BCh into the NE is not inhibited (Figures 9A–D,G).

The non-vesicular mode of uptake of sterols can be found in other systems, where it is proposed that the sterols are internalized by specific transporters that are associated with ER–PM MCS (Gatta et al., 2015; Lahiri et al., 2015). Of particular interest is the work of Gatta et al. (2015), where it was shown that internalization of sterols in yeast occurred in a genetic background where all seven oxysterol-binding proteins, also thought to be involved in sterol transport, had been deleted. The sterol transport capability of yeast cells seemed to be conferred, at least in part, by StART-like proteins, since mutations in these proteins inhibited the uptake of sterols. In that work, the internalization of DHE and cholesterol was monitored by the internal formation of sterol esters and delivery to oil bodies. The first appearance of sterol internalization was at 10–20 min. Here, we have shown that internalization can be detected within 5 min of exposure to BCh (Figure 4). Intriguingly, some of the StART-like proteins in plants have a transmembrane spanning domain in the middle of the protein (Gatta et al., 2015), which may form a single hairpin structure in the cytoplasmic leaflet of the ER. Plant ER proteins known to reside in the cytoplasmic leaflet of the ER, i.e., the membrane-bending reticulons (Zurek et al., 2011) and the large GTP-binding protein involved in ER fusion and/or bundling, Root Hair Defective 3 (RHD3) (Ueda et al., 2016), have a double-hairpin structure. If these StART-like proteins associate with these other proteins on the cytoplasmic side of the ER and are localized to ER–PM MCS, they are excellent candidates for a sterol transporter in plants. In fact, it has recently been shown that one of the reticulons localized to the outer membrane of the ER in plants, RTN20, changes sterol dynamics (Kriechbaumer et al., 2018).

**FIGURE 10:**
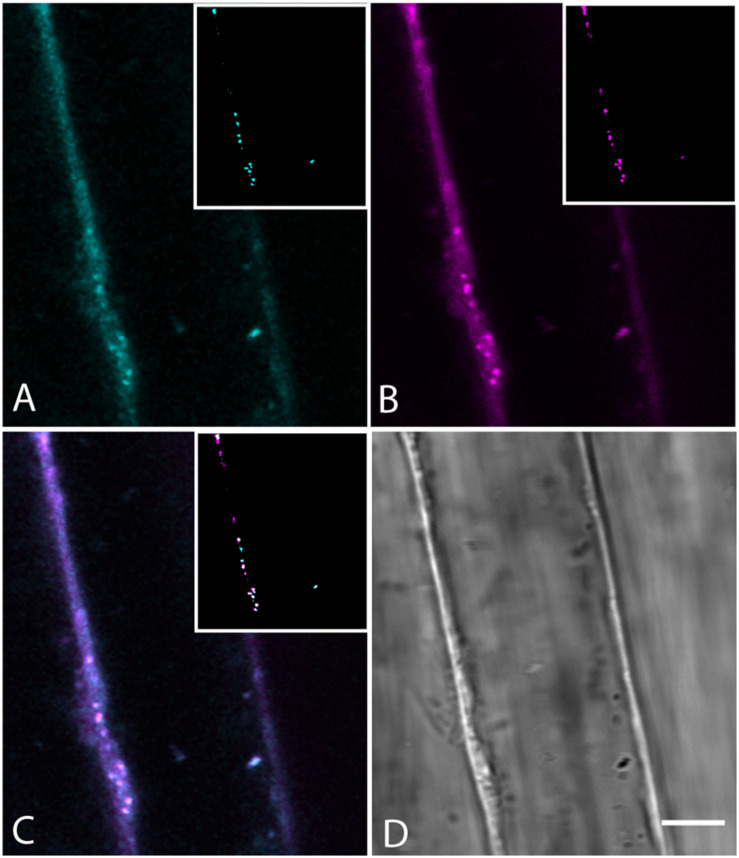
Colocalization of BCh in Nile Red-labeled oil bodies in elongating root cells labeled for 30 min with both probes. **(A)** Fluorescence of BCh at the PM and in the cytoplasm. The inset is a cyan binary mask of the brightest organelles in the image (>128 of 256 gray levels) in the region of the cell shown. **(B)** Fluorescence of Nile Red at the PM and cytoplasm. The inset is a magenta binary mask of the brightest organelles in the image (>128 of 256 gray levels) in the region of the cell shown and marks the oil bodies. **(C)** Merged image of **(A** and **B)**, where the inset is a composite mask of the insets in **(A** and **B)**, showing that the majority of bright organelles are white, or colocalized. **(D)** Bright-field micrograph of the cell. Scale bar = 5 μm.

The compartment of neutral lipids, the oil body, is a well-known place in plants for the storage of lipids and modified sterols and presumably originate from the ER. Overproduction of sterols produces oil bodies containing higher levels of sterol esters (Gondet et al., 1994). Sterol homeostasis is mediated by phospholipid sterol acyltransferase 1, and plants deficient in this enzyme do not accumulate as much lipid in oil bodies (Bouvier-Nave et al., 2010). BCh colocalization with oil bodies (Figure 10) indicates that oil bodies take up and store the extra sterol. The observation is consistent with the model that oil body sterol esters, as in yeast, arise from the ER and are stored inside the oil bodies (Gatta et al., 2015).

## Data Availability Statement

The original contributions presented in the study are included in the article/Supplementary Material, further inquiries can be directed to the corresponding author.

## Author Contributions

LG and KK designed and carried out the experiments. HG and AY carried out the multiphoton microscopy experiments with assistance from LG and KK. LG wrote the manuscript. All authors contributed to the article and approved the submitted version.

## Conflict of Interest

The authors declare that the research was conducted in the absence of any commercial or financial relationships that could be construed as a potential conflict of interest.

## Supplementary Material

Supplementary Figure 1 Absence of growth effects on seedlings grown in the presence of BCh. **(A)** Plate of untreated *Arabidopsis* seedlings. **(B)** Plate of *Arabidopsis* seedlings grown in the presence of 10 μM BCh. **(C)** Root length measured over 3, 5, and 7 days after planting (DAP). **(D)** Hypocotyl length measured over 3, 5, and 7 DAP. diamonds, control; squares, BCh treated.
