# Supplementary figures and images for: The Sterol Trafficking Pathway in Arabidopsis thaliana
Source: Front Plant Sci. 2021 May 26;12:616631. doi: 10.3389/fpls.2021.616631 (PMC8187924; doi:10.3389/fpls.2021.616631)

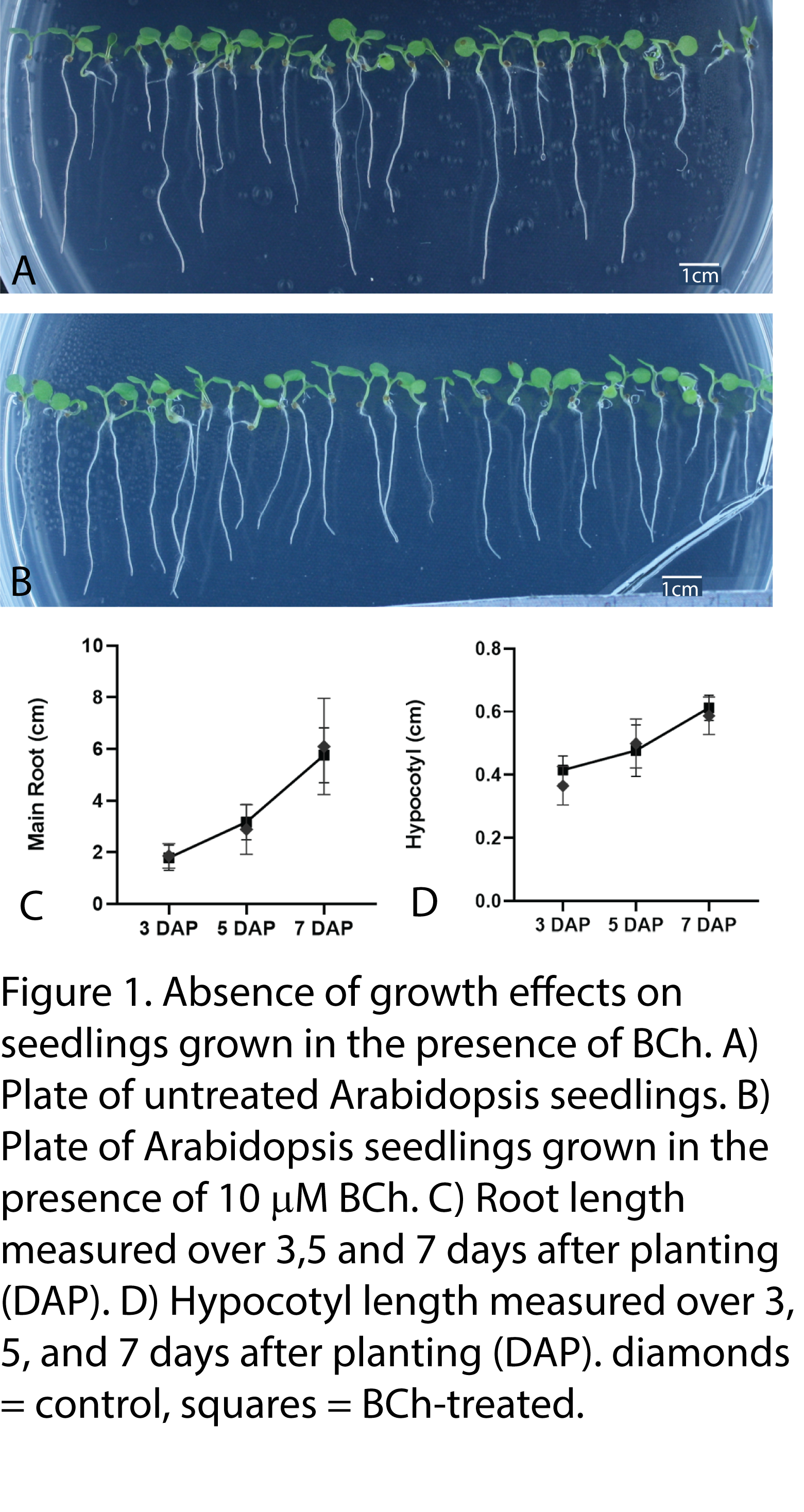

Supplement: Supplementary Figure 1 — Absence of growth effects on seedlings grown in the presence of BCh. (A) Plate of untreated Arabidopsis seedlings. (B) Plate of Arabidopsis seedlings grown in the presence of 10 μM BCh. (C) Root length measured over 3, 5, and 7 days after planting (DAP). (D) Hypocotyl length measured over 3, 5, and 7 DAP. diamonds, control; squares, BCh treated. [file Image_1.TIF]
